# Supplementary material for: A phenotypic in vitro assay of mouse oocyte maturation identifies reversible blockers of meiotic progression for non-hormonal contraceptive discovery
Source: Hum Reprod Open. 2026 May 29;2026(3):hoag051. doi: 10.1093/hropen/hoag051 (PMC13290471; doi:10.1093/hropen/hoag051)
Supplement: hoag051_Supplementary_Data [file hoag051_supplementary_data.docx]

**A phenotypic *in vitro* assay of mouse oocyte maturation identifies reversible blockers of meiotic progression for non-hormonal contraceptive discovery**

Jeffrey Pea, Yiru Zhu, Hoi Chang Lee, Lauren R. Haky, John Proudfoot, Richard Nelson, Francesca E. Duncan

**Supplementary Figures**

Supplementary Figure S1. Filtering strategy of bioactives compound library for compounds screened in oocyte maturation assay.

Supplementary Figure S2. Treatment with nuisance compounds in phenotypic oocyte maturation screening assay.

Supplementary Figure S3. Treatment with HSP90 inhibitors in phenotypic oocyte maturation screening assay.

**Supplementary Figure S1. Filtering strategy of bioactives compound library for compounds screened in oocyte maturation assay.** Selected compounds from the Selleckchem Bioactives Compound Library II were filtered based upon (1) undesirable chemical qualities, (2) undesirable targets, and (3) prioritization based on criteria of target and chemical diversity.

**Supplementary Figure S2. Treatment with nuisance compounds in initial phenotypic oocyte maturation screening assay.** (**A**) Representative images of oocyte phenotypes observed within initial screening assay with treatment of nuisance compounds. Arrows indicate the extruded polar body. (**B**) Table and bar graph of meiotic maturation status of oocytes (n=24 per compound) treated with nuisance compounds during *in vitro* maturation. DMSO, dimethyl sulfoxide; GV, germinal vesicle; GVBD, germinal vesicle breakdown; MII, meiosis II; PAINS, pan-assay interference compounds.

**Supplementary Figure S3. Treatment with HSP90 inhibitors in phenotypic oocyte maturation screening assay.** (**A**) Table of meiotic maturation status of oocytes (n=15-30 per compound) treated with HSP90 inhibitors. (**B**) Bar graph of meiotic maturation status of oocytes (n=15-30 per compound) treated with HSP90 inhibitors. The GV-arrested hit compound identified from the compound screening pipeline (WAY-296993) was treated in parallel as a comparison. DMSO, dimethyl sulfoxide; GV, germinal vesicle; GVBD, germinal vesicle breakdown; MII, meiosis II.
